# Effect of Irradiation on Structural Changes of Levan

**DOI:** 10.3390/ijms23052463

**Published:** 2022-02-23

**Authors:** Dorota Chelminiak-Dudkiewicz, Aleksander Smolarkiewicz-Wyczachowski, Katarzyna Wegrzynowska-Drzymalska, Marta Ziegler-Borowska

**Affiliations:** Medicinal Chemistry Research Group, Department of Biomedical Chemistry and Polymer Science, Faculty of Chemistry, Nicolaus Copernicus University in Torun, Gagarina 7, 87-100 Torun, Poland; 291065@stud.umk.pl (A.S.-W.); kasiawd@doktorant.umk.pl (K.W.-D.)

**Keywords:** levan, photodegradation, irradiation, LED, polysaccharides

## Abstract

Levan, as a biocompatible and renewable biopolymer with anticancer properties, is a promising candidate for a wide range of applications in various fields of industry. However, in the literature, there is a lack of information about its behavior under the influence of UV irradiation, which may limit its potential application, including medical science. Therefore, this study describes the effects of irradiation on the structural properties of levan. This type of fructan was subjected to stability tests under radiation conditions using LED and polychromatic lamps. The results showed that the photodegradation of levan irradiated with a polychromatic light occurs faster and more efficiently than the photodegradation of levan irradiated with an LED lamp. Furthermore, AFM analysis showed that the surface became smoother after irradiation, as evidenced by decreasing values of roughness parameters. Moreover, UV irradiation causes the decrease of total surface free energy and both its components in levan; however, more significant changes occur during irradiation of the sample with a polychromatic lamp.

## 1. Introduction

**Figure 1:**
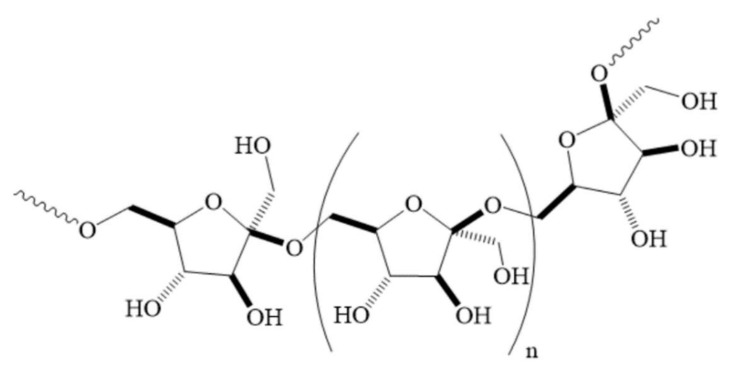
Structure of levan.

Levan is a polysaccharide produced from a few plant species and several microorganisms, such as bacteria, fungi, and yeast. It is an exopolysaccharide whose main chain consists of D-fructofuranosyl groups connected by β-(2,6) bonds, while the branches of the main chain are generated by β-(1,2) bonds (Figure 1) [1,2]. This type of fructan varies in molecular weight and degree of branching, depending on the type of organism from which it is produced. Microbial levans generally have molecular weights over 500,000 Da and are multiply branched, while levan produced by plants often have molecular weights ranging from about 2000 to 33,000 Da [3,4].

Because of levan’s properties (such as biocompatibility, renewability, and eco-friendliness), it is a promising candidate for a wide range of applications in biomedicine, agriculture, as well as food (as a flavor and color stabilizer) and cosmetics (in the preparation of skin creams, moisturizers, etc.) industries [5,6,7,8,9]. Many studies in the literature focus on its anticancer, antioxidant, hypocholesterolemic, and antidiabetic activities [10,11]. It is also used in many different products, including biodegradable plastics, textile coatings, adhesives, and detergents. The wide application of levan in numerous industry sectors has contributed to reducing its production costs.

The structure and morphology of levan can be characterized using many techniques, such as Nuclear Magnetic Resonance Spectroscopy (NMR), Fourier Transforms Infrared Spectroscopy (FTIR), Ultraviolet-Visible Spectroscopy, Raman Spectroscopy, X-ray Diffraction, and Mass Spectroscopy. The stability of levan is determined by its thermal analysis, and its properties, such as tensile strength, Young’s modulus of elasticity, stress, etc., characterize the mechanical strength of this polymer [12,13].

Products based on (bio)polymers (including levan) are exposed to many physical factors. From a practical point of view, the photochemical stability of polymer-based materials is essential, especially when they have to be used at higher temperatures or are exposed to electromagnetic radiation. The degradation of polymers is very complicated. It is influenced by numerous factors, such as temperature, light, atmosphere, humidity, internal and external contamination, and thermal or photochemical catalysts.

In the available literature, there are many studies concerning the degradation (including photodegradation) of various polymers, such as poly (lactic acid) [14] and biopolymers (chitosan, starch, dextran, etc.) [15,16,17]. Still, none of them contains information about the photodegradation of levan. The main factors limiting the application of levan in different fields, including medical sciences, are the lack of results concerning its photochemical stability. Therefore, the present study describes the effects of irradiation on the structural properties of levan. This biopolymer was subjected to stability tests using polychromatic and LED lamps under radiation conditions. The first type of source is beneficial for virus inactivation and bacterial disinfection. On the other hand, LED light provides diverse treatment options, allowing customization for light therapy that can treat brain tumors and fit into balloon catheters intraoperatively while also helping treat minimally invasive procedures such as implanting a flexible LED catheter into a tumor percutaneously. Changes in the chemical structure of the photodegraded levan were determined by FTIR spectroscopy. The surface of the levan before and after exposure was characterized by Scanning Electron Microscopy (SEM), Atomic Force Microscopy (AFM), and contact angle measurement using two measuring liquids. A mechanism of photodegradation of this fructan was also proposed.

## 2. Results and Discussion

### 2.1. Effect of UV Irradiation

The properties of any material depend primarily on its durability and degradation. Moreover, the type of material affects its degradation behavior. The overall reactions during sample irradiation can lead to accelerated decomposition or a stabilizing effect. Today, the irradiation of material, especially polymers, is a popular method for modifying their properties. It leads to chain cleavage and aggregation, double bond formation, and molecular emission. As a result, the properties of the polymer can change, contributing to the material’s applicability [18,19]. Therefore, it is essential to know its radiation behavior.

In this work, two types of lamps were used to study the effect of irradiation on structural changes of levan. The first is a polychromatic lamp (PCh lamp) that emits radiation containing short-term UV. The application of such a lamp is connected with its bactericidal action. Therefore, it is commonly used to sterilize various materials used in the biomedical and pharmaceutical industries and the food industry [20]. The second is a light-emitting diode (LED), which emits only a desired wavelength. In addition, LED consume less energy than high-pressure lamps and tend to be relatively inexpensive in the long term [21]. The UV-Vis spectra of these lamps are included in the Supplementary file (Figure S1). The levan samples were exposed to a high-pressure mercury-vapor light and light-emitting diode, and the changes in their chemical structure were determined by FTIR spectroscopy.

**Figure 2:**
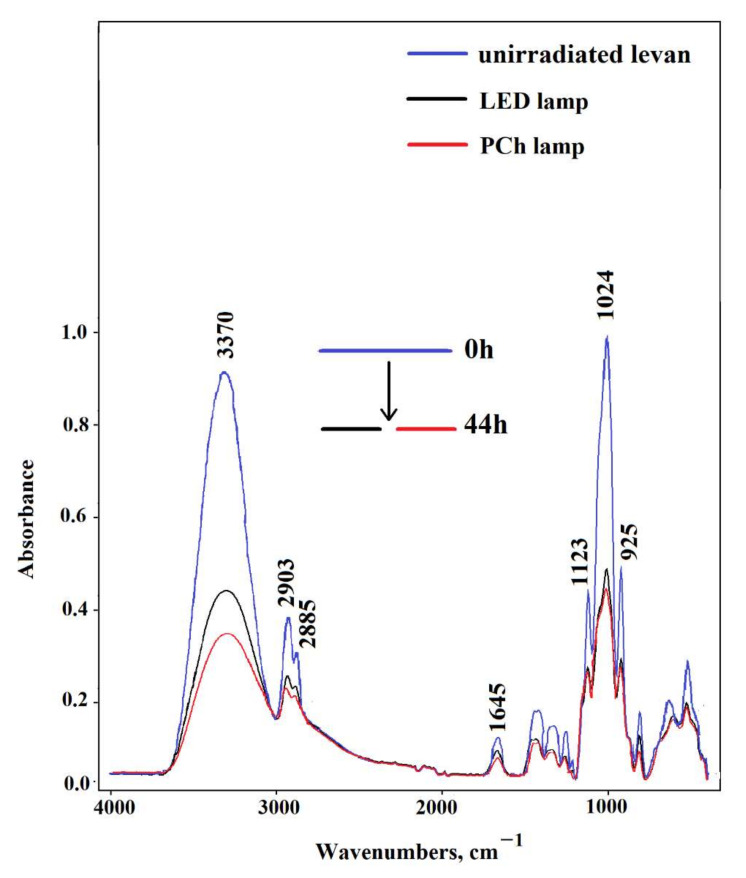
FTIR spectrum for levan films before and after UV irradiation with polychromatic and LED lamps.

FTIR spectroscopy is a valuable technique for analyzing the structure of compounds and the chemical changes on their surface induced by UV irradiation. A detailed analysis of the IR spectra before and after levan’s irradiation with the two types of radiation is shown in Figure 2. The spectra of unirradiated biopolymer show the bands assigned to the OH stretching vibration (3370 cm^−1^) of the polysaccharides and the two bands of C-H stretching vibration (2903 cm^−1^, 2885 cm^−1^), which indicate the existence of fructose residue [22]. Moreover, the bands of stretching vibrations C-O-C (1123 cm^−1^) and C-O-H (1024 cm^−1^) from glycosidic bonds and the bands around 930 cm^−1^ assigned to the stretching vibration of the pyran ring are also observed [9]. The band at 1645 cm^−1^ corresponds to the scissor vibrations from adsorbed water. After drying to constant weight, the water content in the sample was about 9%.

**Figure 3:**
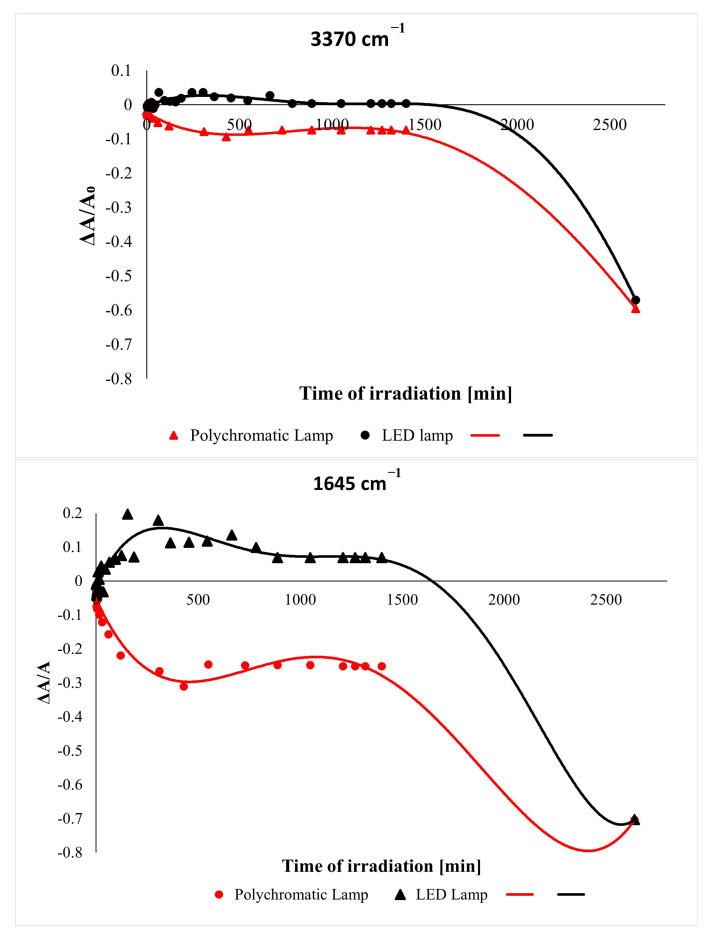

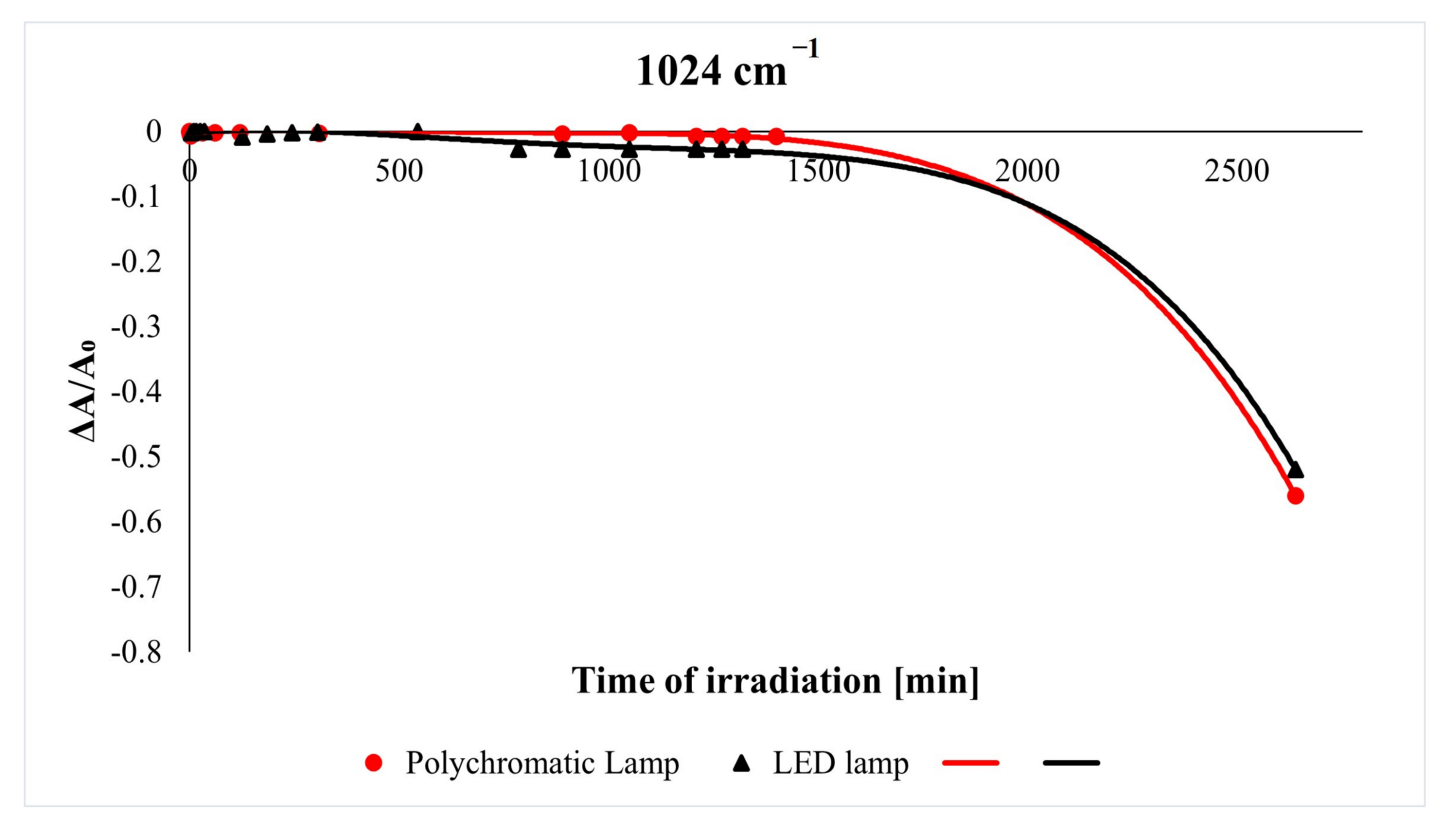
Changes in the relative absorbance of the selected bands of UV-irradiated levan film versus irradiation time.

It was stated that levan was sensitive to both LED and polychromatic light. UV irradiation of samples caused a gradual decrease in all absorption bands of biopolymers. The main changes are observed in the range of the hydroxyl bond vibrations in the spectra of irradiated samples (3370 cm^−1^). This is associated with the loss of absorbed water and the abstraction of OH groups from macromolecules. Evident changes also occur in the bound at 1645 cm^−1^ and 1024 cm^−1^. To graphically present the absorbance decrease at these bonds, the differential parameter was calculated (expressed as the difference of absorbance after a specific irradiation time from the absorbance before irradiation), and then a graph of the dependence of the differential parameter on the irradiation time was plotted (ΔA = f(t)) (Figure 3).

As we can see, the absorbance values of the levan sample gradually decrease and increase. In both cases, the degradation process of the biopolymer is relatively fast in the first 600 min (10 h) of exposure. After about 13 h of irradiation of the sample with an LED lamp and about 16 h of irradiation with polychromatic light, the changes are no longer relevant. The sample was then irradiated for 24 h to each of the lamps, and then the spectrum was recorded. In both cases, the absorbance decreased significantly compared to the unirradiated levan film, but more significant changes were observed after irradiation with a polychromatic lamp. One can conclude that the photodegradation of levan irradiated with an iridescent light occurs faster and more efficiently than the photodegradation of levan irradiated with an LED lamp. Importantly, levan-based materials are unstable under prolonged LED lamp irradiation. This may be related to the microbial origin of this polysaccharide. Most known photodegradable polymers are either toxic to living cells or require intensive UV irradiation for photodegradation. The sensitivity of levan to visible light may provide a new view on its properties, which has application and commercial importance.

### 2.2. SEM Images of Levan Films

**Figure 4:**
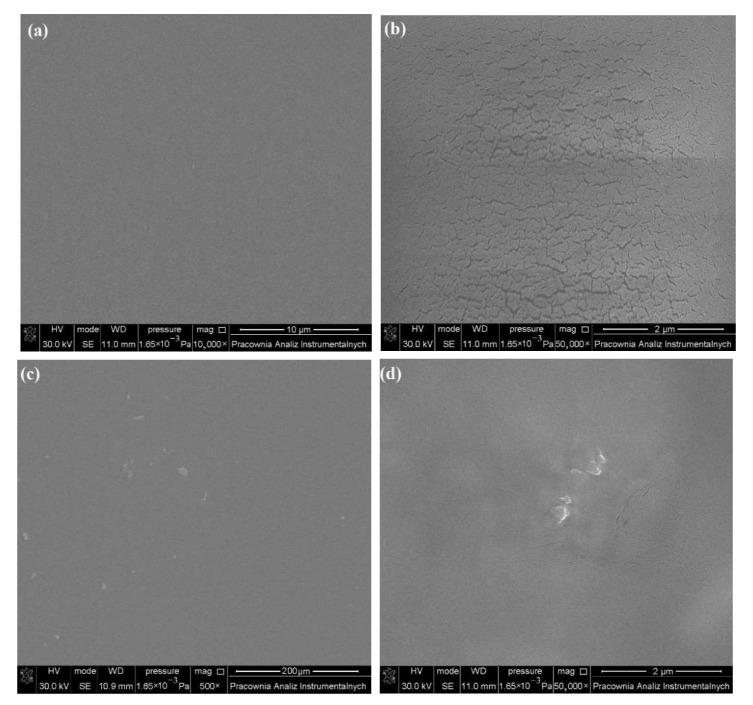
SEM images of the surface of levan films before irradiation (**a**) and after 44 h of irradiation with polychromatic lamp (**b**) and LED lamp (**c**,**d**).

Before irradiation, the levan sample showed a regular and smooth surface without significant defects in cracks and holes (Figure 4a). However, SEM image of the film of levan irradiated with a polychromatic lamp (Figure 4b) shows drastic changes in its surface morphology. The surface of the sample is irregular, and there are numerous microcracks. On the other hand, Figure 4c,d shows mild changes in the surface of the levan film due to the action of LED irradiation. Small microcracks and bubbles appear on the sample surface. According to the literature [23,24], under the influence of irradiation, free radical reactions are generated on the surface, which leads to degradation and/or crosslinking of the polymer. Hence, microcracks and other irregularities appear on the sample surface.

### 2.3. AFM Images of Levan Films

**Figure 5:**
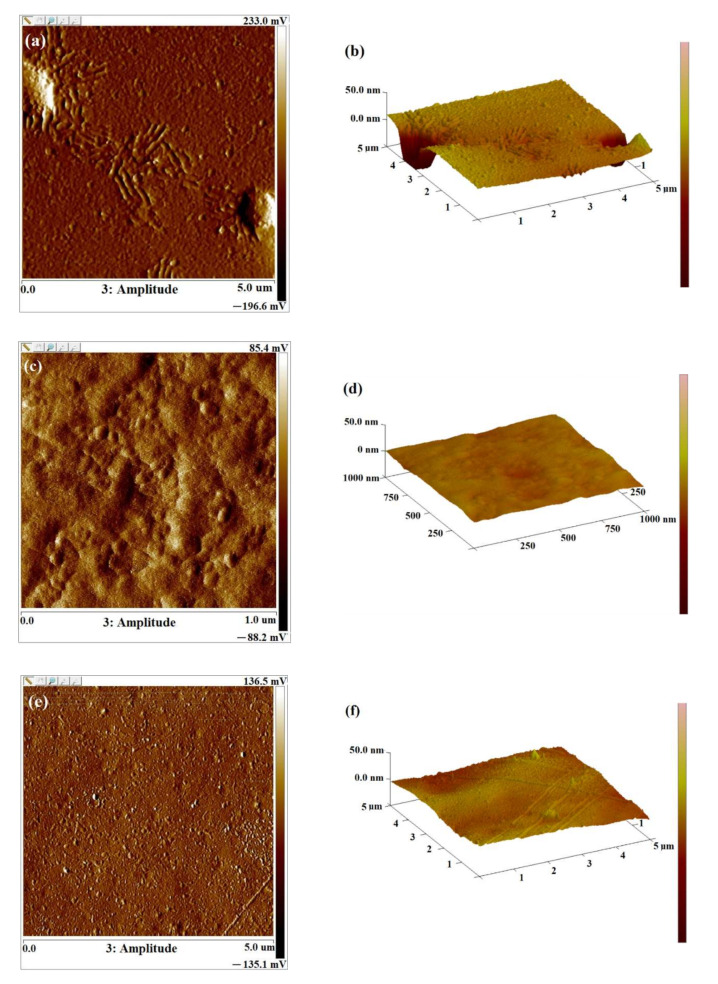
AFM images of the surface of levan films before irradiation (**a**,**b**) and after 44 h of irradiation with a polychromatic lamp (**c**,**d**) and an LED lamp (**e**,**f**).

The atomic force microscopy (AFM) images in Figure 5 exhibit the surface morphology and roughness of levan film and its changes after exposure to UV irradiation. As we can see, the surface of un-irradiated biopolymer is rough and presents structural unity. However, the surface of the levan becomes smoother after irradiation with both a polychromatic lamp and an LED lamp, as evidenced by decreasing values of roughness parameters (Table 1). Moreover, irradiation with a polychromatic lamp for 44 h causes about six times lower roughness parameters than the unirradiated sample. The etching effect of irradiation can explain the drop in roughness parameters. Probably the least orderly sample fragments are photodegraded into volatile products removed from the surface [20]. The reduction of surface roughness can also be due to the water evaporation during irradiation of levan films.

### 2.4. Contact Angle Measurement

Information about the surface properties of levan can affect its practical application. Therefore, the contact angle of the sample was measured, and the results were used to calculate the values of surface free energy and its polar (γ^s^_p_) and dispersive (γ^s^_d_) components of levan film by the Owen-Wendt method [25]. The measurements were performed for levan films before and after exposure to UV using both polychromatic lamp (PCh lamp) and LED lamp, and the results are presented in Table 2.

Levan exhibits a relatively hydrophobic nature, which is confirmed by the higher dispersive component (γ_s_^p^) than the polar component (γ_s_^d^) (Table 2). UV irradiation causes a decrease of total surface free energy and both its components in levan; however, more significant changes occur during irradiation of the sample with a polychromatic lamp. After 44 h of irradiation with the PCh lamp, the surface free energy for levan decreased almost twice. The drop in these values may be caused by the destruction of the biopolymer associated with the removal of polar functional groups from the sample surface.

The obtained results of the polar component values confirm the changes observed in the FTIR spectra in the vibration range of hydroxyl bonds associated with the abstraction of OH groups from macromolecules. Furthermore, the decrease in the content of polar groups on the surface of the biopolymer after irradiation with two types of lamps may be due to a photooxidation reaction during which new carbonyl groups are formed.

### 2.5. Discussion of Photodegradation Mechanism

The photodegradation process occurs in chromophore groups, which are necessary to absorb incident radiation. Polymers are usually unsaturated structures, such as carbonyl, ethylene, or aromatic groups. These chromophores can initiate new chain reactions upon prolonged irradiation and thus rapid destruction of the polymers [26].

**Figure 6:**
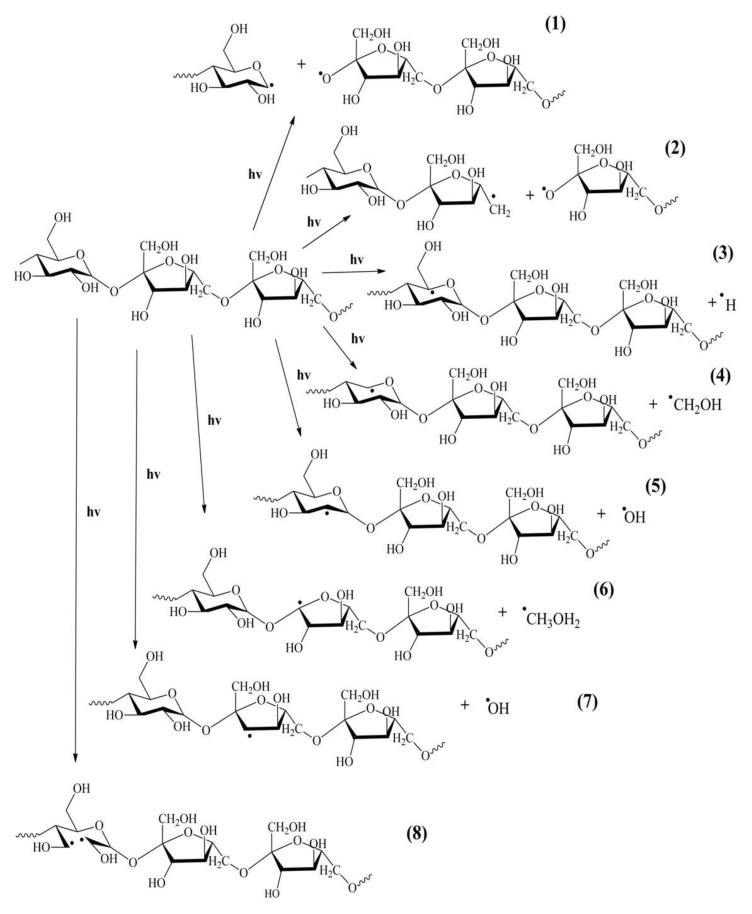
Main photochemical reactions in the levan.

**Figure 7:**
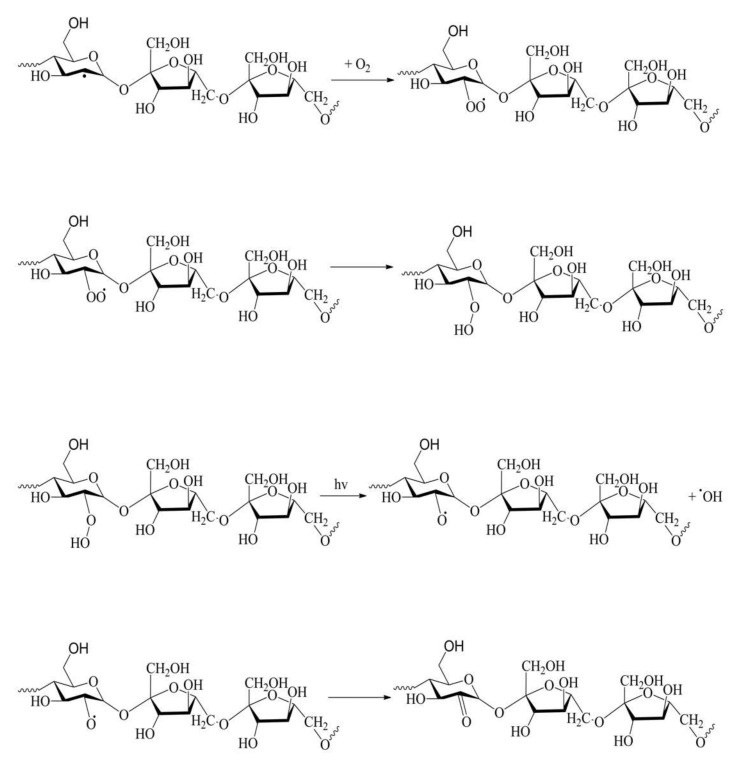
The photo-oxidation process of one of the levan radicals is secondarily formed during irradiation.

In general, photodegradation induced by light exposure of the polymers occurs by Norrish reactions, photoionization (Norrish I) and chain scission (Norrish II). Furthermore, typical nucleophilic addition can occur if suitable reagents (such as water) are present in the reaction medium. The elementary photochemical reactions in levan include the scission of the main chain at the β-2,6 glycosidic bond and the cleavage of side groups (Figure 6). These reactions result in small radicals, such as ·OH, CHO, and CH_2_OH attaching adjacent substituents. The resulting macroradicals react with O_2_ to form peroxy radicals. The increase in band intensity at 1645 cm^−1^ may indicate the appearance on the sample’s surface of C=O bonds, whose presence is characteristic of photo-oxidation processes. Figure 7 shows an example of possible transformations with oxygen leading to a product containing a carbonyl group. In addition, the decrease in peak intensity at about 925 cm^−1^ indicates the breakdown of the ring structure in the levan molecules. Under the influence of UV irradiation of levan, there is also a loss of physically loosely adsorbed water and water more strongly bound to macromolecules, leading to the formation of double bonds. These bonds are responsible for UV absorption and yellowing of irradiated samples. The dehydration process may also cause a decrease in the surface roughness parameters of the irradiated sample. On the other hand, the reduction in the surface roughness of levan can be attributed to the photochemical oxidation process mentioned above. Moreover, UV radiation can generate free radical reactions that lead to the formation of surface irregularities in the form of microcracks and vesicles observed on scanning microscope images. Those radicals may also form a cross-linking structure and branching one, which change the surface of biopolymers. In addition, the spectroscopic results show that the decrease in the polar component after irradiation can be attributed to the photooxidation reaction and the release of polar groups previously linked by hydrogen bonding.

Under UV irradiation, there are changes in the chemical structure of macromolecules and the formation of low molecular weight degradation products [27]. Secondary reactions can occur differently due to the presence of several active intermediates. Deactivation of all radicals leads to the termination of the process. This happens mainly through radical recombination or disproportionation reactions.

## 3. Materials and Methods

### 3.1. Materials and Sample Preparation

Levan from *Erwinia herbicola* (molecular weight 1.3 × 10^6^ Da), glycerin, and diiodomethane (pure for analysis) were purchased from Sigma-Aldrich (Munich, Germany).

To obtain the levan films, pure biopolymer (0.5 g) was dissolved in water (50 mL) and poured into quartz plates to evaporate the solvent. The sample was dried under ambient conditions for 24 h. To determine the water content in the sample, the levan film was dried at 100 °C to a constant weight.

### 3.2. Methods

#### 3.2.1. Photostability Studies

The levan film was exposed to a high-pressure mercury vapor lamp HPK 125 W (Philips, Holland), emitting polychromatic irradiation in the range of 248–578 nm, in an air atmosphere. The radiation intensity was 71 W/m^2^, 53 W/m^2^ and 18.3 W/m^2^ for UVA, UVB, and UVC, respectively, and was measured with a radiometer HD 9021 (Delta, Turin, Italy) at the sample level, i.e., at 10 cm distance from the source. The maximum time of exposure was 44 h. The procedure was tripled. The second lamp used for studies on the photostability of levan was a light-emitting diode (LED) (PrevaLED^®^ Core G7 Food and Fashion LED modules OSRAM, 242, Munich, Germany), emitting radiation at an absorption maximum of 615 nm. The intensity of the emitted radiation was equal to 0.21 μW/cm^2^ and was measured with an electronic radiometer IL 1400A (Newburyport, MA, USA) at the sample level, i.e., at 10 cm distance from the source. The maximum time of exposure was 44 h. The procedure was tripled. The course of the photodegradation of levan has been monitored with FTIR spectroscopy.

#### 3.2.2. Characterization of Levan Films

The thickness of the levan film (measured with a caliper) was 0.0531 mm. The changes in irradiated samples were studied by the FTIR Spectrum-Two™ spectrophotometer (Perkin Elmer, Waltham, MA, USA). Spectra were recorded in the range of 4000–450 cm^−1^, at a resolution of 4 cm^−1^, 32 scans at room temperature.

The surface morphology of levan before and after irradiation was investigated by a scanning electron microscope, SEM (1430VP, LEO Scanning Microscopy Ltd., Cambridge, England) and atomic force microscopy, AFM (MultiMode Nanoscope IIIa Veeco Metrology Inc., USA) techniques. The intermittent mode was applied in AFM. Roughness parameters, arithmetic mean, R_a_, root mean square, R_q_, and the highest peak value, R_max_, were calculated for a 5 × 5 μm^2^ scanning area.

The hydrophilic/hydrophobic properties of levan were analyzed by contact angle (Θ) measurements using a DSA G10 goniometer (Kruss GmbH, Hamburg, Germany) equipped with a camera. A drop of glycerin or diiodomethane was placed on the biopolymer film surface. The producer supplied the software for drop shape analysis and calculation of surface free energy (γs). Each contact angle value is an average of at least ten measurements.

## 4. Conclusions

In summary, the levan film was obtained and tested for its behavior under the influence of UV irradiation. Two lamps were used for the study: a polychromatic lamp and an LED lamp. The results showed that the photodegradation of levan irradiated with a polychromatic light occurs faster and more efficiently than the photodegradation of levan irradiated with an LED lamp. UV irradiation causes a decrease of total surface free energy and both its components in levan; however, more significant changes occur during irradiation of the sample with a polychromatic lamp. Moreover, the photooxidation of levan resulted in crack formations and a decrease in roughness. A mechanism accounting for the main degradation routes for sample photooxidation was also proposed. The results obtained may contribute to the increasing potential applications of levan.

## Figures and Tables

**Table 1:** Roughness parameters (R_q_, R_a_, and, R_max_, nm) of levan films before and after irradiation.

Time of Irradiation [h]	Roughness Parameters [nm]					
	LED Lamp			PCh Lamp		
	R_q_	R_a_	R_max_	R_q_	R_a_	R_max_
0	10.00	4.90	101.0	10.00	4.90	101.0
13	5.45	4.41	57.7	4.19	3.31	34.4
44	3.63	2.97	37.6	1.95	1.47	14.7

**Table 2:** Surface free energy for levan films before and after irradiation.

Time of Irradiation [h]	LED Lamp					PCh Lamp				
	Average Contact Angle [θ, °]		Surface Free Energy [mJ/m^2^]			Average Contact Angle [θ, °]		Surface Free Energy [mJ/m^2^]		
	Glycerin	Diiodomethane	γ_s_	γ_s_^d^	γ_s_^p^	Glycerin	Diiodomethane	γ_s_	γ_s_^d^	γ_s_^p^
0	73.4	71.1	45.33	28.61	16.72	73.4	71.1	45.33	28.61	16.72
13	67.3	66.5	37.21	21.90	15.31	65.6	63.9	33.76	19.23	14.53
44	55.7	53.8	26.35	15.46	10.89	53.1	52.2	21.78	12.44	9.34

## Data Availability

Not applicable.

## Supplementary Materials

The following supporting information can be downloaded at: https://www.mdpi.com/article/10.3390/ijms23052463/s1. References [28,29] are cited in the supplementary materials.
