# Supplementary material for: Effect of Irradiation on Structural Changes of Levan
Source: Int J Mol Sci. 2022 Feb 23;23(5):2463. doi: 10.3390/ijms23052463 (PMC8910695; doi:10.3390/ijms23052463)
Supplement: Supplementary file 1 [file ijms-23-02463-s001.zip › ijms-1596526-supplementary.pdf]

Effect of Irradiation on Structural Changes of Levan

Supplementary Materials

Dorota Chełminiak-Dudkiewicz *, Aleksander Smolarkiewicz-Wyczachowski, Katarzyna Węgrzynowska-Drzymalska and Marta Ziegler-Borowska *

Department of Biomedical Chemistry and Polymer Science, Medicinal Chemistry Research Group, Faculty of Chemistry Nicolaus Copernicus University in Torun, Gagarina 7, 87-100 Torun, Poland

* Correspondence: MZB; martaz@umk.pl, DChD: dorotachd@umk.pl

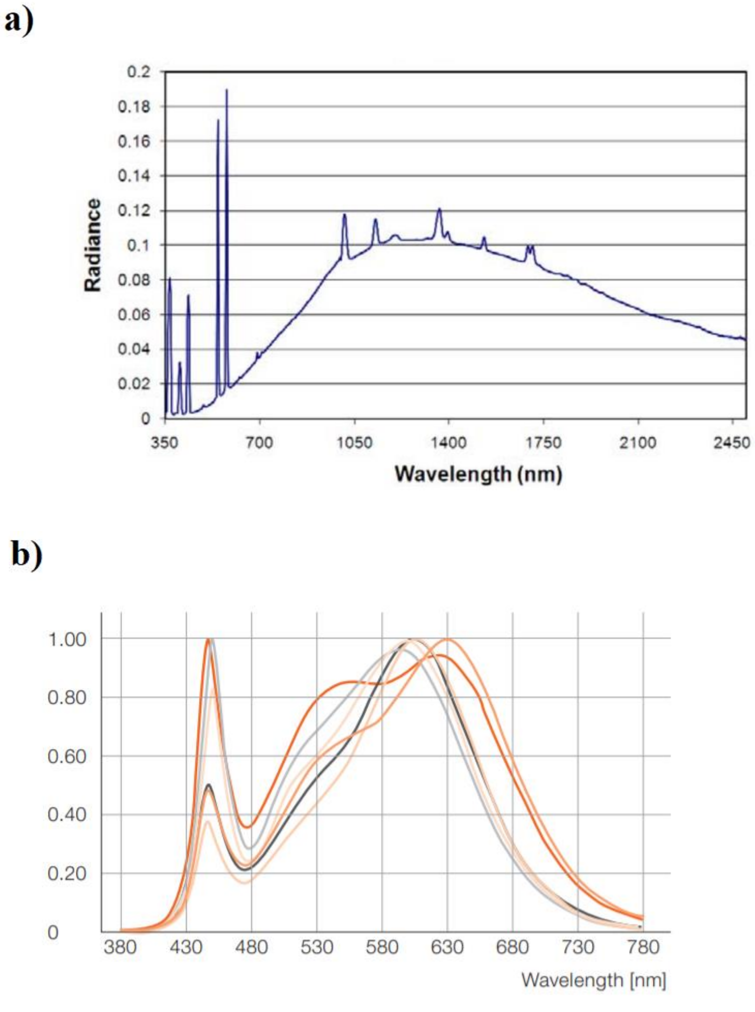

Figure S1. Spectrum of the (**a**) polichromatic [1] and (**b**) LED lamps [2].

References

[1]. Elvidge, C.D.; Keith, D.M.; Tuttle, B.T.; Baugh, K.E. Spectral Identification of Lighting Type and Character. *Sensors* **2010**, *10*, 3961–3988. https://doi:10.3390/s100403961.

[2]. Technical Application Guide. PrevaLED® Core Z6 LED Modules. Available online: www.osram.com/prevaled-core (access on 16 July 2020).
